# Quality of Life in Patients with Hepatic Encephalopathy Treated with Rifaximin: A Systematic Review

**DOI:** 10.3390/jcm15145600

**Published:** 2026-07-16

**Authors:** Rodolfo Sacco, Ambra Corti, Luca Giacomelli, Antonio Facciorusso

**Affiliations:** 1 Gastroenterology and Digestive Endoscopy Unit, Department of Surgical and Medical Sciences, University of Foggia, Viale Pinto 1, 71122 Foggia, Italy; 2 Polistudium SRL, 20121 Milan, Italy; ambra.corti@polistudium.it (A.C.); luca.giacomelli@polistudium.it (L.G.); 3 Gastroenterology Unit, Department of Experimental Medicine, Università del Salento, 73100 Lecce, Italy; antonio.facciorusso@virgilio.it

**Keywords:** hepatic encephalopathy, rifaximin, quality of life, cirrhosis

## Abstract

**Background/Objectives:** Hepatic encephalopathy (HE) significantly impairs quality of life (QoL) in patients with cirrhosis. While rifaximin is established in HE treatment and prevention, its specific impact on QoL remains less clearly defined. This systematic review aims to evaluate the effects of rifaximin on QoL in patients with HE. **Methods:** A comprehensive literature search was conducted in MEDLINE/PubMed, Embase, and CENTRAL through November 2025. Clinical studies evaluating rifaximin’s impact on QoL in patients with cirrhosis and minimal/covert or recurrent overt HE were included. Study selection followed PRISMA guidelines. Data extraction focused on study design, population, treatment, and QoL outcomes. **Results:** Out of 4343 records screened, 10 studies met the inclusion criteria. Several studies evaluated rifaximin in comparison with placebo and focused on patients with minimal or covert HE. Rifaximin was generally associated with improvements in overall and domain-specific QoL scores compared with placebo. These benefits were observed across different dosages and treatment durations. In comparative studies, rifaximin showed QoL outcomes comparable to those of lactulose, L-ornithine L-aspartate, and combination regimens. **Conclusions:** Rifaximin may be associated with improvements in QoL in patients with cirrhosis and HE, with benefits observed across multiple domains and outcomes broadly comparable to alternative therapies. However, given the limitations of available evidence and the heterogeneity in study designs, HE phenotypes, QoL instruments, and outcome reporting, these findings should be interpreted with caution. Further high-quality comparative trials, particularly in patients with recurrent overt HE and in prophylactic settings, are needed to confirm these findings.

## 1. Introduction

Hepatic encephalopathy (HE) is a neuropsychiatric complication commonly observed in patients with liver cirrhosis [1]. It results from impaired hepatic detoxification or portosystemic shunting, leading to the systemic accumulation of neurotoxins—primarily ammonia—which alters cerebral functions [2].

Clinically, HE presents along a spectrum from mild cognitive and psychomotor disturbances to severe manifestations, such as unconsciousness and cerebral edema [3]. It is broadly classified into covert HE, which includes minimal and grade I presentations, and overt HE, defined by more evident neurological symptoms of grade II or higher [3,4].

The management of HE primarily aims to reduce systemic ammonia levels. In addition, systemic inflammation, endotoxemia and gut microbiota-driven immune activation are increasingly recognized as contributors to HE pathophysiology [5]. Lactulose, a nonabsorbable disaccharide, remains a first-line therapy for episodic overt HE and is frequently used to prevent recurrence. It functions by decreasing ammonia generation and absorption in the gut, effectively reducing systemic ammonia levels [3]. Despite its efficacy, its gastrointestinal side effects, such as bloating, abdominal cramping and diarrhea, may compromise patients’ adherence [6].

Rifaximin, a non-systemic oral antibiotic, is an effective option, either alone or in combination with lactulose, for the treatment of HE [7]. It targets the gut microbiota, and specifically ammonia-producing bacteria, leading to reduced ammonia generation and absorption [2]. In addition, it seems to play a role in gut barrier repair, potentially reducing bacterial translocation and systemic endotoxemia in patients with cirrhosis [8]. A placebo-controlled trial showed that rifaximin was effective in treating overt and covert HE, decreased gut bacterial overgrowth and systemic inflammation in patients with cirrhosis and HE [8]. Rifaximin has also been shown to reduce circulating levels of inflammatory cytokines, including IL-6, IL-10 and TNF-α, in patients with alcoholic cirrhosis, nonalcoholic fatty liver disease-related cirrhosis, and HE [9]. Lastly, rifaximin’s minimal systemic absorption makes it well-tolerated and suitable for long-term use [6].

Clinical trials and meta-analyses have shown that rifaximin is at least as effective as lactulose in improving HE symptoms. When used in combination, the two agents may provide superior outcomes, including higher resolution rates, lower mortality and shorter hospital stays [1,3,7]. Current guidelines support rifaximin mainly as an add-on therapy to lactulose for secondary prophylaxis after recurrent overt HE, whereas evidence for primary prophylaxis remains limited and inconsistent [10]. Long-term observational data have reported sustained clinical benefits with rifaximin, including reductions in ammonia levels and stabilization of liver function, without adverse effects on renal parameters [4].

Although HE is potentially reversible with appropriate treatment, the condition is still associated with poor prognosis and significant quality of life (QoL) impairment [1]. Even minimal HE is linked to cognitive decline, psychomotor slowing, sleep disturbances, and behavioral changes that impair daily functioning, increase the risk of falls or injury and may interfere with complex activities such as driving [2,4]. Additionally, HE imposes a significant emotional and psychological burden on caregivers, contributing to distress, uncertainty, and reduced QoL [1].

### Aim of the Review

This systematic review aims to critically assess the current literature on the effects of rifaximin therapy on QoL in patients with cirrhosis and HE. Notably, a previous systematic review has evaluated patient-reported outcomes in patients with HE treated with lactulose and/or rifaximin [11]. The present review complements this work by focusing specifically on rifaximin, used alone or in combination, and by providing an updated synthesis of QoL outcomes through November 2025.

## 2. Methods

### 2.1. Search Strategy

A comprehensive literature search was conducted up to 20 November 2025, using the following databases: MEDLINE/PubMed (National Library of Medicine), Embase (Ovid), and the Cochrane Central Register of Controlled Trials (CENTRAL). The search string contained Thesaurus Terms (Medical Subject Headings/MeSH for PubMed and Emtree Terms for Embase) and their synonyms (free-text terms), combined using the Boolean operators (AND–OR). The search strategy can be found in the Supplementary Material File S1.

**Figure 1:**
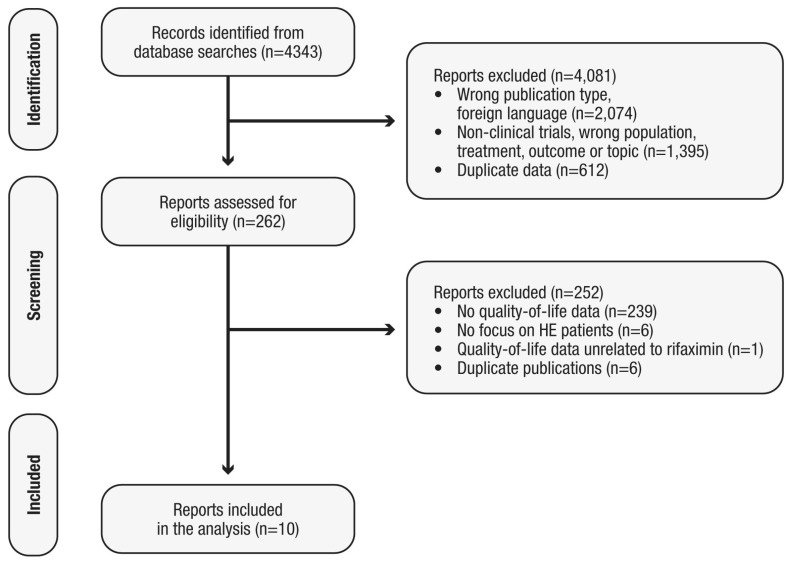
PRISMA flowchart representing the study selection process.

References were managed using RefWorks-ProQuest, and Rayyan software was used to identify and remove duplicates. This was done to ensure data integrity and improve transparency of the process and results. The Preferred Reporting Items for Systematic Reviews and Meta-Analyses (PRISMA) methodology was used to select studies, and the PRISMA flowchart (Figure 1) summarizes the Search and Screening Process of this study. The PRISMA 2020 checklist is provided in the Supplementary Materials (Table S1).

### 2.2. Eligibility Criteria

During the first phase of screening, titles and abstracts were reviewed and excluded if they met one of the following criteria: (1) the article was not written in English; (2) the article was a review, case report, letter, editorial, cost-effectiveness analysis, non-clinical study; (3) the trial was terminated or withdrawn; (4) the trial did not evaluate rifaximin or HE and QoL; and (5) the trial evaluated rifaximin for other indications other than cirrhosis. The inclusion criteria were as follows: (1) clinical trials, including randomized, non-randomized, observational, retrospective and uncontrolled studies; (2) studies investigating QoL or QoL-related outcomes; and (3) studies including subjects with cirrhosis and minimal/covert or recurrent overt HE regardless of age, sex, etiology of condition, or presence of other precipitating factors. The selection phase was performed using Rayyan Software.

### 2.3. Data Extraction

For all articles included in the first phase of screening, the following data were extracted: authors, title, year, ISSN, volume, issue, pages, DOI, abstract and keywords.

### 2.4. Data Selection

A total of 262 full-text articles and abstracts were identified and reviewed. Two authors independently screened the titles and abstracts of retrieved records to assess their relevance to the research objective. Publications presenting overlapping or duplicate study data were also examined, and only the most comprehensive or most recent version was retained. Any disagreements regarding study selection were resolved through discussion until consensus was reached.

The following data were extracted for all included articles: authors, year, study design, population, treatment(s), QoL assessment and QoL findings.

### 2.5. Risk of Bias Assessment

The methodological quality and risk of bias of the included studies were assessed using design-specific tools. Randomized controlled trials were evaluated using the Cochrane Risk of Bias 2 tool, which assesses bias across five domains: randomization process, deviations from intended interventions, missing outcome data, outcome measurement, and selection of reported results. Observational studies were assessed using the Newcastle–Ottawa Scale, which evaluates study quality across the domains of selection, comparability, and outcome assessment. Each study was classified as having high or low overall quality based on the results of the corresponding assessment tool.

## 3. Results

### 3.1. Characteristics of the Selected Studies

The database search identified 4343 studies (816 from MEDLINE/PubMed, 2500 from EMBASE, and 1027 from CENTRAL). After the removal of 612 duplicate records, 3731 unique articles were screened by title and abstract. Of these, 3469 studies were excluded according to the inclusion/exclusion criteria. A total of 262 full-text articles and abstracts were assessed for eligibility, while 125 records could not be retrieved (Figure 1).

After full-text screening, 252 articles were excluded. The main reasons were the absence of quality-of-life data (e.g., guidelines, healthcare professional surveys, consensus papers, presentation of study design without reported results, studies focusing on quality of care or cognitive outcomes; *n* = 239); lack of focus on patients with HE (*n* = 6); quality-of-life data not related to rifaximin treatment (*n* = 1); and duplicate publications of the same study (*n* = 6). Overall, 10 studies met the inclusion criteria and were included in the systematic review. The study selection process is summarized in Figure 1.

Six studies investigated the impact of rifaximin on QoL without active comparator therapies (Table 1) [12,13,14,15,16,17], while four directly compared rifaximin with alternative treatment strategies, including lactulose, L-ornithine L-aspartate (LOLA), nitazoxanide, or combination regimens (Table 2) [18,19,20,21]. Notably, two studies—Bajaj et al. and Bruyneel et al.—primarily focused on cognitive function and driving performance, and on sleep quality, respectively [14,15]. However, as both included QoL outcomes, they were retained for analysis.

The majority of studies were randomized controlled trials, either double-blind (*n* = 5) or open-label (*n* = 2), while three were observational studies. Most studies enrolled patients with minimal or covert HE (*n* = 7), while three focused on individuals with recurrent overt HE.

### 3.2. Risk of Bias Assessment

The methodological quality and risk-of-bias assessment of the included studies are summarized in Table 3. Most randomized controlled trials were judged to be of overall high quality. Two studies were rated as low quality, mainly due to concerns related to randomization and deviations from intended interventions. Among observational studies, two were rated as high quality according to the Newcastle–Ottawa Scale, while one was rated as low quality. Overall, the available evidence was considered acceptable, although heterogeneity in study design and methodological quality should be considered when interpreting the findings.

### 3.3. Summary of QoL Outcomes

Among the six studies without active comparators, three were randomized controlled trials, one was a randomized open-label study, and two were observational. Sample sizes ranged from 15 to 258 participants, with treatment durations between 8 weeks and 12 months.

Rifaximin was associated with improvements in overall QoL, most commonly assessed by the Chronic Liver Disease Questionnaire (CLDQ) and the Sickness Impact Profile (SIP). Improvements were reported across multiple QoL domains, with superior outcomes compared with placebo for total SIP scores and for all CLDQ domains, including fatigue, abdominal symptoms, systemic symptoms, activity, emotional function, and worry. In one study, no significant difference was observed between low-dose (800 mg/day) and high-dose (1200 mg/day) rifaximin over 8 weeks, although the relatively short follow-up may have limited the ability to detect dose-related differences in patient-reported outcomes [16]. Notably, rifaximin dosages of 550 mg twice daily and 1200 mg/day are the reference regimens most consistently associated with clinically meaningful outcomes in patients with HE, including reductions in recurrence rates, hospital readmissions, and mortality, as reported in pivotal randomized trials and long-term observational studies [22,23,24].

Two studies did not report significant improvements in overall QoL. In Bajaj et al. [14], among patients with minimal/covert HE, total and most domain-specific SIP scores were not significantly different between rifaximin and placebo; however, a significant improvement was observed in the psychosocial domain after 8 weeks of rifaximin. Bruyneel et al. also found no significant changes in QoL in recurrent over HE based on the Short Form-36 (SF-36) or Hospital Anxiety and Depression Scale (HADS) [15].

The four comparative studies focused mainly on patients with minimal HE. Compared with lactulose, rifaximin showed comparable improvements in QoL outcomes, with no statistically significant differences in total or domain-specific scores [18,21]. Studies comparing rifaximin with multiple therapeutic strategies, including lactulose and LOLA, showed similar QoL improvements [19,20]. In one head-to-head trial versus nitazoxanide in patients with recurrent overt HE, rifaximin was associated with domain-specific improvements in QoL, particularly in fatigue and activity. In contrast, nitazoxanide demonstrated a greater overall improvement in CLDQ scores [20].

## 4. Discussion

This systematic review summarizes the available evidence on the impact of rifaximin on QoL in patients with cirrhosis and HE.

In studies without active comparators, rifaximin was associated with statistically significant improvements in overall QoL in some analyses, particularly in patients with minimal or covert HE. QoL benefits were reported across multiple domains, including fatigue, activity, emotional function, abdominal symptoms, systemic symptoms and worry [12,13]. These effects were documented primarily using SIP and CLDQ, providing a degree of methodological consistency across studies. Two studies did not report significant improvements in overall QoL with rifaximin. However, both were limited by small sample sizes (*n* = 42 and *n* = 15, respectively) and were not primarily designed to assess QoL. In Bajaj et al., rifaximin did not significantly improve total SIP or most subdomain scores, but significantly improved the psychosocial domain. This was accompanied by significant improvements in cognitive test performance and driving simulator outcomes compared with placebo [14]. Similarly, Bruyneel et al. found no significant changes in standard QoL or sleep questionnaires (SF-36, HADS, PSQI and EES); however, objective sleep assessments showed significant improvements in REM sleep duration and overall sleep architecture as measured by polysomnography. Notably, this study was judged to be of low quality in the risk-of-bias assessment [15].

Comparative studies generally reported no significant differences in QoL outcomes between rifaximin and other therapies, such as lactulose, LOLA and combination regimens [18,19,21]. A single trial comparing rifaximin with nitazoxanide reported greater improvement in CLDQ scores with nitazoxanide, while rifaximin demonstrated significant domain-specific benefits in fatigue and activity [20]. Given the small sample size of this study, these findings should be interpreted cautiously.

Taken together, these findings suggest that rifaximin may improve QoL in selected patients with cirrhosis and HE, particularly those with minimal or covert HE; however, the clinical meaningfulness of these changes remains difficult to establish. The heterogeneity of QoL findings may be explained by differences in HE severity, rifaximin regimens, follow-up duration, outcome instruments, and study methodology. SIP, CLDQ, SF-36, and SF-12 assess different QoL dimensions, limiting direct comparison across studies. In addition, open-label design, small sample size, variable risk of bias, and the use of QoL as a secondary or exploratory endpoint may have contributed to inconsistent results. Notably, three of the ten studies were judged to be of low quality, which may have affected the estimated magnitude and consistency of the observed QoL benefits. Overall, these factors suggest that the evidence should be interpreted as supportive but not definitive.

Economic considerations are also relevant when interpreting the potential role of rifaximin in HE management. Although rifaximin is more expensive than lactulose, available pharmacoeconomic evidence suggests that rifaximin, particularly in combination with lactulose for the secondary prevention of overt HE, may be cost-effective by reducing recurrence, hospitalizations, and length of inpatient stay [1]. However, further cost analyses incorporating primary care resource use are warranted.

Further comparative research is needed to clarify the impact of rifaximin and alternative therapies on QoL in patients with HE, as current evidence is insufficient to support a preference for one treatment over another. Future studies should focus on patients with recurrent overt HE and on prophylactic use, settings in which evidence is currently limited. A multicentre, randomized, double-blind, placebo-controlled trial is currently investigating the efficacy and safety of rifaximin and lactulose in patients with cirrhosis undergoing placement of a transjugular intrahepatic portosystemic shunt, with QoL assessed using the LDSI v2.0 and EQ-5D-5L questionnaires. Results from this trial are not yet available [25].

### Strengths and Limitations

This systematic review has several strengths. Most included studies were randomized controlled trials [12,13,17,20] and risk-of-bias assessment indicated that the majority were of acceptable methodological quality. In addition, most of the studies used validated and similar QoL instruments, namely the SIP and CLDQ, thus supporting the comparability of the observed QoL effects.

This review should be interpreted alongside the previous systematic review and meta-analysis by Moon et al. [11], which assessed patient-reported outcomes in HE and included both rifaximin and lactulose. Although this overlap may limit the novelty of our work, the present review provides a rifaximin-focused and more updated synthesis of data.

Several limitations should be acknowledged. Despite the considerable number of articles initially retrieved, only a limited number of studies fulfilled the inclusion criteria, particularly those providing direct comparisons between rifaximin and alternative therapies. the limited number of studies together with the relatively small number of total enrolled patients may limit the generalizability of results. Most studies focused on patients with minimal or covert HE, whereas data on recurrent overt HE were limited. In addition, most studies evaluated rifaximin as a treatment strategy rather than for prophylaxis [16,18]. Although liver-associated outcomes such as hospitalization, infections, recurrent HE, and cirrhotic decompensation events may indirectly influence QoL, these outcomes were not systematically extracted or analyzed because the review focused on QoL assessed through validated questionnaires or scales. In addition, the exclusion of 423 non-English articles may have introduced language bias and limited the comprehensiveness of the review. Finally, two studies were available only in abstract format, limiting the completeness of methodological reporting and outcome detail [19,21].

A formal meta-analysis was not performed because the included studies were heterogeneous in design, HE phenotype, treatment regimen and duration, and QoL tools. Although several studies used the same tool (SIP or CLDQ), QoL results were reported in non-uniform formats across studies, such as total scores, selected domain-specific scores, within-group baseline-to-follow-up changes, and between-group comparisons. This variability limited the derivation of a common effect estimate suitable for pooling. Accordingly, forest plots and a funnel plot were not generated. Lastly, this systematic review was not registered in PROSPERO or another prospective registry.

## 5. Conclusions

This systematic review suggests that rifaximin may be associated with QoL improvements in patients with cirrhosis and HE, particularly in minimal or covert HE. Reported benefits were measured using validated instruments and were registered across multiple domains relevant to daily functioning and well-being. These findings complement existing evidence on the clinical efficacy of rifaximin in HE management by emphasizing its impact on patient-centered outcomes.

However, these findings should be interpreted with caution. The available evidence is limited by the small number of studies, modest sample sizes, heterogeneous study designs and QoL measures and limited methodological quality of some of the studies.

Future well-designed randomized trials are needed to clarify the impact of rifaximin on QoL in patients with both covert and overt HE, as well as in both prophylactic and treatment settings.

## Figures and Tables

**Table 1:** Summary of studies on the impact of rifaximin on quality of life in hepatic encephalopathy.

Author(s), Year	Study Design & Population	Treatment	QoL Tools	Main QoL Findings
Minimal/Covert HE				
Sidhu et al., 2011 [12] (RIME trial)	RCT, double blind; *n* = 94	RFX 1200 mg/day (*n* = 49) vs. placebo (*n* = 45) Duration: 8 weeks	SIP	Total SIP: RFX: 11.67 → 6.45, *p* < 0.001; placebo: 9.86 → 8.51; *p* = 0.82. Correlates: improvement in psychometric tests
Bajaj et al., 2011 [14]	RCT, double blind; *n* = 42	RFX 550 mg BID (*n* = 21) vs. placebo (*n* = 21) Duration: 8 weeks	SIP; driving simulation and cognitive testing	SIP psychosocial: RFX 13 ± 3 → 8 ± 2, *p* = 0.04; placebo 13 ± 4 → 11 ± 3, *p* = 0.45 Other SIP dimensions: NS Driving errors: RFX −76% vs. placebo −33%, *p* = 0.013. Cognition: RFX 91% vs. placebo 61%, *p* = 0.02.
Tan et al., 2022 [16]	RCT, open label, *n* = 40	RFX low dose: 800 mg/day (*n* = 12) RFX high dose: 1200 mg/day f (*n* = 14) Duration: 8 weeks Control (*n* = 14)	SIP	Total SIP: low dose vs. control: −1.98 vs. 0; *p* = 0.048; high dose vs. control: −4.00 vs. 0; *p* = 0.007. Low vs. high dose: NS, *p* = 0.592.
Bakulin et al., 2023 [17]	Observational, *n* = 258	Continuous: 1200 mg/day for 12 months (*n* = 41) Cyclic: 600–1200 mg/day for 7–14 days/month (*n* = 217)	CLDQ	Total CLDQ: improved in both subgroups. Between subgroups: NS.
**Recurrent overt HE**				
Sanyal et al., 2011 [13]	RCT, double blind, *n* = 219	RFX 550 mg BID (lactulose permitted) (*n* = 101) vs. placebo (*n* = 118) Duration: 6 months	CLDQ	Overall CLDQ: RFX vs. placebo, *p* = 0.0093. Domains improved: fatigue *p* = 0.0087; abdominal symptoms *p* = 0.0090; systemic symptoms *p* = 0.0160; activity *p* = 0.0022; emotional function *p* = 0.0065; worry *p* = 0.0436.
Bruyneel et al., 2017 [15]	Observational, *n* = 15	RFX 550 mg BID + daily lactulose Duration: 28 days	SF-36, HADS	SF-36/HADS: NS. REM sleep: 2.5%→8.5%, *p* = 0.003. REM + stage 3 sleep: 37.6%→55.6%, *p* = 0.007.

BID: twice a day; CLDQ: Chronic Liver Disease Questionnaire; HADS: Hospital Anxiety and Depression Scale; HE: hepatic encephalopathy; NS: non-significant; REM: Rapid Eye Movement; RFX: rifaximin; SF-36: Short Form-36 Health Survey; SIP: Sickness Impact Profile.

**Table 2:** Summary of studies on the impact of rifaximin versus comparators on quality of life in hepatic encephalopathy.

Author(s), Year	Study Design & Population	Treatment Regimens Compared	QoL Tools	Main QoL Findings
Minimal/Covert HE				
Sidhu et al., 2016 [18]	RCT, open label, *n* = 112	RFX 400 mg TID (*n* = 57) vs. lactulose 30–120 mL/day (*n* = 55) Duration: 3 months	SIP	**Total SIP**: RFX15.7 → 7.5 vs. lactulose: 15.2 → 8.2; adjusted mean difference: −0.92 (95% CI −2.36–0.52; *p* = 0.20) **SIP domains**: NS.
Elnoemany et al., 2017 [19]	Observational; *n* = 126	Lactulose 30–60 mL BID (*n* = 31) vs. RFX 200 mg TID (*n* = 32) vs. LOLA 6 g TID (*n* = 32) vs. combination therapy (*n* = 31)	SIP	**Total SIP improvement**: 24.7 vs. 16.1 vs. 16.3; *p* = 0.001 **Between groups**: NS; *p* > 0.05.
Sahid Aziz et al., 2023 [21]	RCT, open label; *n* = 31	RFX 400 mg TID (*n* = 15) vs. lactulose 30–120 mL/day (*n* = 16) Duration: 12 weeks	SF-12 (PCS, MCS); PSQI	**SF-12 physical/mental scores**: improved in both groups; *p* < 0.001. **PSQI**: improved at 12 weeks; *p* < 0.001. **Between groups**: NS for QoL outcomes.
Recurrent overt HE				
Glal et al., 2021 [20]	RCT, double blind; *n* = 60	RFX 550 mg BID (*n* = 30) vs. nitazoxanide 500 mg BID (*n* = 30) Duration: 24 weeks	CLDQ	**Total CLDQ**: RFX: 3.5 ± 0.7 → 3.6 ± 0.9, *p* < 0.39; nitazoxanide: 3.6 ± 0.7 → 4 ± 0.3, *p* = 0.0004 RFX improvement in CLDQ domains: fatigue *p* = 0.013; activity *p* = 0.019.

BID: twice a day; HE: hepatic encephalopathy; LOLA: L-Ornithine L-Aspartate; RFX: rifaximin; SIP: Sickness Impact Profile; TID: thrice a day.

**Table 3:** Risk of bias assessment and quality of included studies.

Observational Studies ^a^	Selection		Comparability	Outcome		Overall Quality
Bakulin et al., 2023 [17]	***		**	**		H
Bruyneel et al., 2017 [15]	**		NA	**		L
Elnoemany et al., 2017 [19]	*		*	*		H
**Randomized Controlled Trials ^b^**	**1**	**2**	**3**	**4**	**5**	**Overall Quality**
Sahid Aziz et al., 2023 [21]	U	U	H	L	L	L
Bajaj et al., 2011 [14]	L	L	L	L	L	H
Glal et al., 2021 [20]	L	L	L	L	L	H
Sanyal et al., 2011 [13]	L	L	L	L	L	H
Sidhu et al., 2011 [12]	L	U	H	L	L	L
Sidhu et al., 2016 [18]	L	L	L	L	L	H
Tan et al., 2022 [16]	L	L	L	L	L	H

^a^ Study quality assessment performed by means of the Newcastle–Ottawa scale (each asterisk represents whether the respective criterion within the subsection was satisfied). ^b^ Cochrane Collaboration’s tool 2 for assessing the risk of bias across 5 domains: 1 (Random sequence generation), 2 (Deviations from the intended interventions), 3 (Missing outcome data), 4 (Outcome measurement), 5 (Selection of the reported results). L = low; H = high; U = unclear; NA = not applicable.

## Data Availability

All data are available from the corresponding author upon reasonable request.

## Supplementary Materials

The following supporting information can be downloaded at: https://www.mdpi.com/article/10.3390/jcm15145600/s1, File S1: Search Strategy; Table S1: PRISMA 2020 Checklist [26].
