# Supplementary material for: Quality of Life in Patients with Hepatic Encephalopathy Treated with Rifaximin: A Systematic Review
Source: J Clin Med. 2026 Jul 16;15(14):5600. doi: 10.3390/jcm15145600 (PMC13413256; doi:10.3390/jcm15145600)
Supplement: Supplementary file 1 [file jcm-15-05600-s001.zip › File S1. Supplementary Material.pdf]

Supplementary Material

Search Strategy

The research has been done on three databases: MEDLINE/PubMed (National Library of Medicine), EMBASE (Ovid) and the Cochrane Central Register of Controlled Trials (CENTRAL) until 20 November 2025.

To retrieve all the existing literature about the topic, the search string contains Thesaurus Terms (Medical Subject Headings/MeSH for PubMed and Emtree Terms for Embase) and Emtree terms (for Embase) and their synonymous (free-text terms). A Boolean search approach was followed for searching the databases by combining all the key search terms and synonymous in a group with the "OR" operator and the entire search groups with the "AND" operator.

Any differences between the following Search Strategies are due to the different technical characteristics and content of the databases used: MEDLINE/PubMed is a free, publicly accessible database of biomedical literature maintained by the U.S. National Library of Medicine (NLM). EMBASE is a comprehensive biomedical and pharmacological database containing records with a strong focus on drugs; CENTRAL is a database of bibliographic reports of randomized controlled trials.

MEDLINE/PubMed

#1 "Liver Cirrhosis, Alcoholic"[Mesh] OR "Liver Cirrhosis, Alcoholic"[Title/Abstract] OR "Alcoholic Liver Cirrhosis"[Title/Abstract] OR "Alcoholic Cirrhosis"[Title/Abstract] OR "Hepatic Cirrhosis, Alcoholic"[Title/Abstract] OR "Alcoholic Hepatic Cirrhosis"[Title/Abstract]

#2 "Liver Cirrhosis"[Mesh] OR "cirrhosis"[Title/Abstract] OR "decompensated cirrhosis"[Title/Abstract] OR "advanced liver disease"[Title/Abstract] OR Cirrho*[Title/Abstract] OR "Liver Cirrhosis"[Title/Abstract]

OR "Cirrhosis, Liver"[Title/Abstract] OR "Hepatic Cirrhosis"[Title/Abstract] OR "Cirrhosis, Hepatic"[Title/Abstract] OR "Fibrosis, Liver"[Title/Abstract] OR "Liver Fibrosis"[Title/Abstract]

#3 "Hepatic Encephalopathy"[Mesh] OR "Hepatic Encephalopathy"[Title/Abstract] OR "Encephalopathies, Hepatic"[Title/Abstract] OR "Hepatic Encephalopathies"[Title/Abstract] OR "Portosystemic Encephalopathy"[Title/Abstract] OR "Encephalopathies, Portosystemic"[Title/Abstract] OR "Portosystemic Encephalopathies"[Title/Abstract] OR "Encephalopathy, Hepatic"[Title/Abstract] OR "Encephalopathy, Hepatocerebral"[Title/Abstract] OR "Encephalopathies, Hepatocerebral"[Title/Abstract] OR "Hepatocerebral Encephalopathies"[Title/Abstract] OR "Encephalopathy, Portal-Systemic"[Title/Abstract] OR "Encephalopathies, Portal-Systemic"[Title/Abstract] OR "Encephalopathy, Portal Systemic"[Title/Abstract] OR "Portal-Systemic Encephalopathies"[Title/Abstract] OR "Encephalopathy, Portosystemic"[Title/Abstract] OR "Hepatocerebral Encephalopathy"[Title/Abstract] OR "Portal-Systemic Encephalopathy"[Title/Abstract] OR "Portal Systemic Encephalopathy"[Title/Abstract] OR "Hepatic Coma*"[Title/Abstract] OR "Coma*, Hepatic"[Title/Abstract] OR "Hepatic Stupor*"[Title/Abstract] OR "Stupor, Hepatic"[Title/Abstract] OR "Stupor*, Hepatic"[Title/Abstract] OR "Fulminant Hepatic Failure with Cerebral Edema"[Title/Abstract]

#4 "Fibrosis"[Mesh] OR Fibrosis[Title/Abstract] OR Fibroses[Title/Abstract] OR Cirrhosis[Title/Abstract]

#5 "Rifaximin"[Mesh] OR "Rifaximin"[Title/Abstract] OR "Redactiv"[Title/Abstract] OR "Xifaxan"[Title/Abstract] OR Xifaxan*[Title/Abstract] OR Rcifax*[Title/Abstract]

#6 #1 OR #2 OR #3 OR #4

#7 #5 AND #6

EMBASE (Ovid)

#1 'hepatic encephalopathy'/exp OR 'ammoniac encephalopathy':ab,kw,ti OR 'encephalopathy, ammoniac':ab,kw,ti OR 'encephalopathy, hepatic':ab,kw,ti OR 'encephalopathy, porta cava':ab,kw,ti OR 'encephalopathy, portacaval':ab,kw,ti OR 'hepato cerebral disease':ab,kw,ti OR 'hepatocerebral disease':ab,kw,ti OR 'hepatocerebral syndrome':ab,kw,ti OR 'hepatoencephalopathy':ab,kw,ti OR 'hepatogenous encephalopathy':ab,kw,ti OR 'liver encephalopathy':ab,kw,ti OR 'porta cava encephalopathy':ab,kw,ti OR 'portacaval encephalopathy':ab,kw,ti OR 'portal encephalopathy':ab,kw,ti OR 'portal systemic encephalopathy':ab,kw,ti OR 'portocaval encephalopathy':ab,kw,ti OR 'hepatic encephalopathy':ab,kw,ti

#2 'liver disease'/exp OR 'hepatic disease':ab,kw,ti OR 'hepatic disorder':ab,kw,ti OR 'hepatopathy':ab,kw,ti OR 'liver cell disease':ab,kw,ti OR 'liver diseases':ab,kw,ti OR 'liver disorder':ab,kw,ti OR 'liver illness':ab,kw,ti OR 'liver disease':ab,kw,ti

#3 'alcohol liver cirrhosis'/exp OR 'alcohol cirrhosis':ab,kw,ti OR 'alcohol liver injury':ab,kw,ti OR 'alcoholic cirrhosis':ab,kw,ti OR 'alcoholic liver':ab,kw,ti OR 'alcoholic liver cirrhosis':ab,kw,ti OR 'alcoholic liver damage':ab,kw,ti OR 'cirrhosis, alcoholic':ab,kw,ti OR

'laennec cirrhosis':ab,kw,ti OR 'laennec's cirrhosis':ab,kw,ti OR 'liver alcohol cirrhosis':ab,kw,ti OR 'liver alcoholic cirrhosis':ab,kw,ti OR 'liver cirrhosis, alcoholic':ab,kw,ti OR 'alcohol liver cirrhosis':ab,kw,ti

#4 'liver cirrhosis'/exp OR 'cirrhosis':ab,kw,ti OR 'cirrhosis hepatis':ab,kw,ti OR 'cirrhosis, liver':ab,kw,ti OR 'cryptogenic liver cirrhosis':ab,kw,ti OR 'dietary cirrhosis':ab,kw,ti OR 'dietary liver cirrhosis':ab,kw,ti OR 'hepatic cirrhosis':ab,kw,ti OR 'postnecrotic liver cirrhosis':ab,kw,ti OR 'liver cirrhosis':ab,kw,ti

#5 'liver fibrosis'/exp OR 'fibrosis, liver':ab,kw,ti OR 'fibrous hepatic disease':ab,kw,ti OR 'hepatic fibrosis':ab,kw,ti OR 'hepato-fibrosis':ab,kw,ti OR 'hepatofibrosis':ab,kw,ti OR 'liver periportal fibrosis':ab,kw,ti OR 'periportal fibrosis':ab,kw,ti OR 'liver fibrosis':ab,kw,ti

#6 'rifaximin'/exp OR 'abimix':ti,ab,kw OR 'colidimin':ti,ab,kw OR 'dermodis':ti,ab,kw OR 'fatroximin':ti,ab,kw OR 'flonorm':ti,ab,kw OR 'l 105 (lepetit)':ti,ab,kw OR 'lormyx':ti,ab,kw OR 'lumenax':ti,ab,kw OR 'normicron':ti,ab,kw OR 'normix':ti,ab,kw OR 'redactiv':ti,ab,kw OR 'refero':ti,ab,kw OR 'rifacol':ti,ab,kw OR 'rifamixin':ti,ab,kw OR 'rifamycin l 105':ti,ab,kw OR 'rifaxamin':ti,ab,kw OR 'rifaxidin':ti,ab,kw OR 'rifaxin':ti,ab,kw OR 'rifxima':ti,ab,kw OR 'spiraxin':ti,ab,kw OR 'targaxan':ti,ab,kw OR 'tixtar':ti,ab,kw OR 'tixteller':ti,ab,kw OR 'xifaxan':ti,ab,kw OR 'xifaxanta':ti,ab,kw OR 'zaxine':ti,ab,kw OR 'rifaximin':ti,ab,kw

#7 #1 OR #2 OR #3 OR #4 OR #5

#8 #6 AND #7

Cochrane Central Register of Controlled Trials (CENTRAL)

#1 MeSH descriptor: [Liver Cirrhosis, Alcoholic] explode all trees

**#2**<br>"Alcoholic Cirrhosis"<br>Cirrhosis"):ti,ab,kw	("Liver Cirrhosis, Alcoholic" OR "Alcoholic Liver Cirrhosis" OR<br>OR "Hepatic Cirrhosis, Alcoholic" OR "Alcoholic Hepatic
**#3**	MeSH descriptor: [Liver Cirrhosis] explode all trees
**#4**<br>disease" OR Cirrho*<br>"Cirrhosis,	("cirrhosis" OR "decompensated cirrhosis" OR "advanced liver<br>OR "Liver Cirrhosis" OR "Cirrhosis, Liver" OR "Hepatic Cirrhosis" OR<br>Hepatic" OR "Fibrosis, Liver" OR "Liver Fibrosis"):ti,ab,kw
**#5**	MeSH descriptor: [Hepatic Encephalopathy] explode all trees
**#6**<br>"Hepatic<br>"Encephalopathies,<br>"Encephalopathy,<br>"Encephalopathies,<br>"Encephalopathy,<br>"Encephalopathy,<br>"Encephalopathy,<br>Systemic<br>Coma" OR<br>"Stupor, Hepatic" OR	("Hepatic Encephalopathy" OR "Encephalopathies, Hepatic" OR<br>Encephalopathies" OR "Portosystemic Encephalopathy" OR<br>Portosystemic" OR "Portosystemic Encephalopathies" OR<br>Hepatic" OR "Encephalopathy, Hepatocerebral" OR<br>Hepatocerebral" OR "Hepatocerebral Encephalopathies" OR<br>Portal- Systemic" OR "Encephalopathies, Portal-Systemic" OR<br>Portal Systemic" OR "Portal-Systemic Encephalopathies" OR<br>Portosystemic" OR "Hepatocerebral Encephalopathy" OR "Portal-<br>Encephalopathy" OR "Portal Systemic Encephalopathy" OR "Hepatic<br>"Coma,Hepatic" OR "Hepatic Stupor" OR "Stupor, Hepatic" OR<br>"Fulminant Hepatic Failure with Cerebral Edema"):ti,ab,kw
**#7**	MeSH descriptor: [Fibrosis] explode all trees
**#8**	("Fibroses" OR "Cirrhosis" OR Fibrosis OR Cirrhosi):ti,ab,kw
**#9**	MeSH descriptor: [Rifaximin] explode all trees
**#10**	(Rifaximin OR Redactiv OR Xifaxan):ti,ab,kw

**#11**	#1 OR #2 OR #3 OR #4 OR #5 OR #6 OR #7 OR #8
**#12**	#9 OR #10
**#13**	#11 AND #12
